# Inhibition of interleukin‐1 with rilonacept is not effective in cold urticaria—Results of a randomized, placebo‐controlled study

**DOI:** 10.1002/clt2.12226

**Published:** 2023-02-28

**Authors:** Hanna Bonnekoh, Monique Butze, Sebastian Spittler, Petra Staubach, Karsten Weller, Jörg Scheffel, Marcus Maurer, Karoline Krause

**Affiliations:** ^1^ Institute of Allergology Charité—Universitätsmedizin Berlin Corporate Member of Freie Universität Berlin Humboldt‐Universität zu Berlin and Berlin Institute of Health Berlin Germany; ^2^ Fraunhofer Institute for Translational Medicine and Pharmacology ITMP, Allergology and Immunology Berlin Germany; ^3^ Autoinflammation Reference Center Charité (ARC2) Charité—Universitätsmedizin Berlin Berlin Germany; ^4^ Klinik für Anästhesiologie, Intensivmedizin, Schmerztherapie und Notfallmedizin Bundeswehrkrankenhaus Berlin Germany; ^5^ Department of Dermatology University Medical Center Mainz Mainz Germany

**Keywords:** autoinflammation, cold urticaria, dermatology, immunology, interleukin‐1, rilonacept, urticaria

## Abstract

**Background:**

Cold urticaria (ColdU) is characterized by pruritic wheals following exposure of the skin to cold. Many patients show insufficient response to antihistamines, the first line treatment. Based on the high efficacy of interleukin‐1(IL‐1)‐inhibition in cold‐induced urticarial autoinflammatory diseases, we assessed the effects of rilonacept, an IL‐1 inhibitor, in ColdU patients unresponsive to standard treatment.

**Methods:**

In this randomized, double‐blind, placebo‐controlled two‐center study, we included 20 patients with ColdU. In the first part, patients received 320 mg rilonacept or placebo (1:1) followed by weekly doses of 160 mg rilonacept or placebo for 6 weeks. In the second part, all patients received weekly 160 mg or 320 mg rilonacept for 6 weeks, open‐label. The primary endpoint was change in critical temperature threshold (CTT). Secondary endpoints included changes in quality of life impairment (Dermatology Life Quality Index, DLQI), differences of inflammatory mediators upon cold provocation and safety assessment over the study period.

**Results:**

Baseline mean CTTs were 20.2°C (placebo) and 17.3°C (rilonacept). Mean CTTs did not change significantly during the 6‐week double‐blind treatment (placebo – 0.45°C; rilonacept +0.89°C). IL‐6, IL‐18 and HSP‐70 blood levels showed interindividual variability without significant changes during hand cold water bath provocation in placebo‐ or rilonacept‐treated patients. In contrast, DLQI significantly improved in the rilonacept (mean DLQI reduction of 3.8; *p* = 0.002) but not in the placebo group (mean DLQI reduction of 0). Comparing baseline with the rilonacept open‐label treatment, there were no changes in CTTs or DLQI scores.

**Conclusion:**

IL‐1 inhibition with rilonacept did not improve ColdU, but demonstrated a good safety profile.

**Clinical Trial Registration:**

EudraCT number: 2012‐005726‐30. ClinicalTrials.gov identifier: NCT02171416.

## 1 BACKGROUND

Acquired cold urticaria (ColdU) is characterized by pruritic wheals and/or angioedema following exposure of the skin to cold. Skin symptoms typically arise minutes after exposure to cold objects, liquids or air and are usually limited to cold‐exposed skin areas.^1^ However, extensive cold exposure for example, swimming in cold water can lead to generalized urticarial symptoms and systemic reactions such as dyspnea, hypotension and loss of consciousness.^2^ In addition, consumption of cold foods and beverages can result in oropharyngeal angioedema.^3^, ^4^ ColdU can, therefore, be a life‐threatening disease.^5^, ^6^

ColdU is the second most prevalent subtype of chronic inducible urticaria with an estimated incidence of 0.05% in Central Europe.^7^ It affects young adults most frequently but may occur at any age.^7^, ^8^ ColdU is a self‐limited disease with approximately 50% of patients experiencing remission after 6 years.^8^

The exact pathogenesis of ColdU is not completely understood. The development of wheals and angioedema is thought to be driven by the activation and degranulation of cutaneous mast cells with subsequent release of various inflammatory mediators including histamine, leukotrienes and cytokines after cold exposure of the skin.^9^, ^10^, ^11^, ^12^

Current treatment strategies in ColdU comprise the avoidance of cold exposure and the use of H1 antihistamines.^13^ Antihistamine treatment in ColdU patients significantly reduces cutaneous histamine release as well as levels of interleukin (IL)‐6, a downstream effector of IL‐1β, and IL‐8 after cold exposure.^14^ However, many patients with ColdU show insufficient response to treatment with antihistamines, even at high doses.^15^, ^16^ The reasons for this are currently unknown.^8^ In antihistamine non‐responders, anti‐IgE treatment with omalizumab may be an effective treatment option.^17^ Nevertheless, omalizumab is not approved for ColdU, and not all ColdU patients treated with omalizumab show improvement. Novel and better treatment options for patients with ColdU are needed, and several are currently being explored or in clinical development.^12^, ^18^, ^19^, ^20^, ^21^

Different systemic autoinflammatory diseases present with cold‐induced wheals. The best known entity is cryopyrin‐associated periodic syndrome (CAPS), for which IL‐1‐targeting drugs have been shown to be highly effective.^22^, ^23^, ^24^ Familial cold autoinflammatory syndrome (FCAS) is a subform of CAPS and characterized by cold‐induced urticarial rash, fever, arthralgia and fatigue.^25^ IL‐1β is the pivotal proinflammatory cytokine in many autoinflammatory diseases and accounts for fever and systemic inflammation. Rilonacept is a dimeric fusion protein that inhibits both, IL‐1α and IL‐1β. It is licensed in the US for the treatment of CAPS including FCAS and Muckle‐Wells‐syndrome (MWS) in adults and children aged 12 years and older. In CAPS patients, rilonacept shows favorable effects on systemic symptoms and cold‐induced urticarial rash.^22^ Of note, anti‐IL‐1 treatment was reported to be beneficial in other chronic inflammatory dermatologic conditions including a patient with acquired ColdU with inadequate symptom control and multifactorial autoinflammatory diseases presenting with wheals such as Schnitzler syndrome and adult‐onset Still's disease (licensed use in Europe for anakinra and canakinumab for the last‐mentioned).^26^, ^27^, ^28^

We hypothesized that patients with acquired ColdU who are unresponsive to antihistamines may benefit from IL‐1 inhibition. Therefore, the objective of this study was to assess the efficacy and safety of rilonacept in patients with ColdU who are not successfully treated with antihistamines.

## 2 METHODS

### 2.1 Study design

This phase II, multi‐center, investigator‐initiated parallel group study was conducted in two parts. After a 2‐week screening period, part A consisted of a 6‐week randomized, double‐blind, placebo‐controlled phase in which the patients were randomized (1:1) to one of two groups: (1) rilonacept or (2) placebo. Patients in the rilonacept group received a loading dose of 320 mg (2 injections of 160 mg), followed by weekly injections of rilonacept 160 mg, subcutaneously. In the open label treatment phase (part B), after unblinding, patients who had received placebo, were treated with rilonacept 160 mg for additional 6 weeks.

Complete rilonacept responders – that is patients without a wheal‐and‐flare reaction in response to provocation testing with 4°C – were treated with 160 mg. Partial responders – defined as patients with reductions of at least 4°C in critical temperature threshold (CTT, detailed explanation of the method see below) – received 320 mg rilonacept weekly doses. Non‐responders—that is patients with CTT increase, maintenance or reduction <4°C to rilonacept – were excluded from the open‐label extension. There was an additional 4‐week follow‐up period to monitor safety.

### 2.2 Patients

Adult patients with symptomatic ColdU, that is, cold‐induced wheals and itch for more than 6 weeks, who were resistant to conventional antihistamine treatment in standard doses were recruited at Urticaria Centers of Reference and Excellence^29^ (UCAREs; Department of Dermatology and Allergy, Charité—Universitätsmedizin Berlin; Department of Dermatology, Universitätsmedizin Johannes Gutenberg‐Universität, Mainz, both Germany). Patients with a positive cold stimulation test at 4°C (assessed by TempTest^®^ 3.0; EMO Systems GmbH, Berlin Germany) were included. The main exclusion criteria were concurrent/ongoing treatment with immunosuppressive medication, ongoing use of IL‐1 blockers or other biologics; H2 antihistamines, leukotriene antagonists and H1 antihistamines other than rescue medication, known hypersensitivity to rilonacept; significant concomitant illness; pregnancy or breast‐feeding; treatment with a live (attenuated) virus vaccine during 3 months prior to randomization, evidence of active, recurrent or latent systemic infection; and a history of malignancies within 5 years before screening. During the course of the study regular intake of antihistamines was not allowed. However, if necessary, patients were allowed to take rescue medication (loratadine, up to a maximum of 4 tablets of 10 mg per day) throughout the study except the 3 days prior to each study visit. Patients were asked to document the intake of the exact amount of rescue medication for each day.

The study received approval from the study center's Institutional Review Board (Landesamt für Gesundheit und Soziales, Ethik‐Kommission des Landes Berlin) (EudraCT number: 2012‐005726‐30; ClinicalTrials.gov identifier: NCT02171416). All patients provided written informed consent, and the study was conducted according to the Good Clinical Practice Guideline, the Declaration of Helsinki and the International Conference on Harmonization.

## 3 CLINICAL ASSESSMENTS

### 3.1 Critical temperature threshold testing

CTT was defined as the highest temperature at which a wheal reaction was induced by testing for 5 min. To assess CTTs, the TempTest^®^ 3.0 was applied directly on the volar surface of the forearm at 4, 6, 8, 10, 12, 14, 16, 18, 20, 22, 24, and 26°C simultaneously for 5 min. The CTT was assessed at each visit for each patient.

Non‐responders to rilonacept treatment and complete responders to placebo treatment were withdrawn from the study after the end of the double‐blind treatment. Subjects who discontinued the study were treated with standard of care by their attending physicians.

### 3.2 Hand cold water bath

Cold provocation by water bath was performed by immersion of one hand in water of 4°C for 5 min, at week 6 and week 12. This was done under continuous supervision of a healthcare professional and in the presence of adequate emergency measures (emergency medication readily available, i. v. access). Before and after water bath cold provocation (time points 0, +5, +10, +20 min), patients were subjected to blood draws from the cubital vein of the provocation arm. Blood samples were stored at −80°C and processed for serum and plasma measurements of mast cell mediators. IL‐1RA (pg/ml), IL‐6 (pg/ml), IL‐18 (pg/ml) and heat shock protein (HSP) 70 (pg/ml) were assessed in the serum of the patients. HSP70 was chosen as it is known to modulate various cellular processes, including protein folding, regulation of signaling pathway, degradation of misfolded proteins and the modulation of immune responses. HSP70 is induced by stressors including temperature changes and plays an essential role in environmental stress tolerance and thermal adaptation and has been shown to be significantly elevated in patients with chronic spontaneous urticaria (CSU).^30^, ^31^

### 3.3 Study endpoints

The primary endpoint was the change in CTT from baseline to week 6 in the rilonacept group as compared to the placebo group.

Secondary endpoints were (1) change in CTTs in ColdU patients from baseline to week 6; (2) Change in the patient's quality of life, as assessed by the Dermatology Life Quality Index (DLQI) from baseline to week 6 in the rilonacept group as compared to the placebo group and from baseline to the open‐label treatment with rilonacept; (3) differences in mast cell mediator release in the blood of ColdU patients during the challenge with cold water at week 6 in the rilonacept group as compared to the placebo group and during the open‐label treatment in the rilonacept 160 mg group as compared to the 320 mg group and (4) safety of rilonacept treatment (160 and 320 mg weekly; including physical examination, routine safety laboratory assessments, vital signs and adverse event [AE] reporting, assessment of serum anti‐rilonacept antibody levels).

### 3.4 Statistics

For continuous variables, the descriptive statistics included: the number of patients for each calculation (s), mean, median, standard deviation, minimum and maximum. For categorical data, frequencies and percentages were given for each category. The paired *t*‐test was used to calculate the significance. *p*‐values ≤0.05 (two‐sided) were considered significant. All analyses were performed with GraphPad Prism 6 and IBM SPSS statistics 27.

## 4 RESULTS

### 4.1 Patients

**FIGURE 1:**
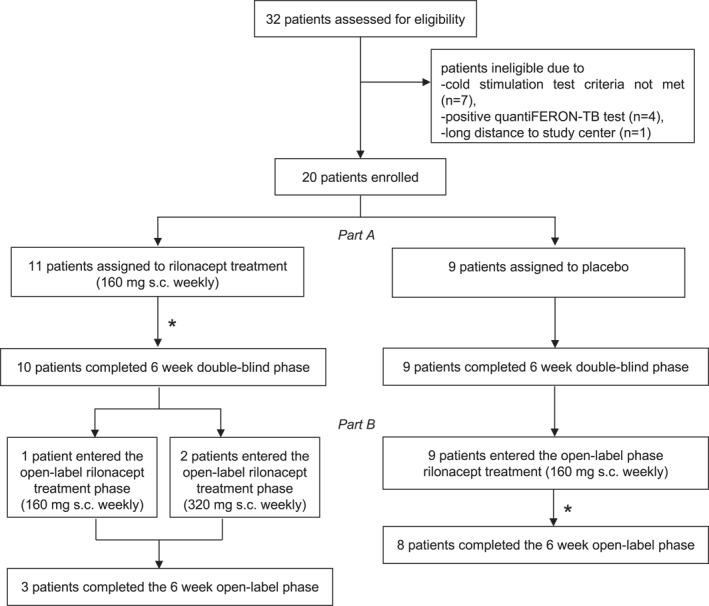
Patient flow (number of screened, enrolled and completed patients). *One patient (rilonacept group) discontinued because of leukocytosis during placebo‐controlled treatment, and one patient discontinued because of loss of efficacy during the open‐label treatment.

In total, 32 patients with ColdU were screened and 20 were randomized for placebo‐controlled treatment with rilonacept (female/male ratio 13:7; mean age 45.6 years, SD 14.2, range 21–72 years; mean duration of ColdU: 156.2 months; Table 1). Nine and 11 patients with ColdU were included in the placebo group and in the treatment group, respectively. Of these, 12 patients (9 placebo‐treated and 3 rilonacept‐treated individuals) transitioned to the open‐label part of the study. Across all patients, 19 and 11 completed the placebo‐controlled and the open‐label treatment part of the study, respectively (Figure 1). One patient (rilonacept group) discontinued because of leukocytosis during placebo‐controlled treatment, and one patient discontinued because of loss of efficacy during the open‐label treatment. The study was conducted from February 2015 until March 2018.

**TABLE 1:** Characteristics of the 20 cold urticaria patients, ColdU Cold urticaria, CTT critical temperature threshold, IQR Interquartile Range, SD Standard deviation.

	All patients (*n* = 20)	Placebo (*n* = 9)	Rilonacept (160 mg; *n* = 11)
Gender			
Male	7 (35%)	5 (56%)	2 (18%)
Female	13 (65%)	4 (44%)	9 (82%)
Mean age (years), IQR	45.55 (33–58)	34 (24–58)	49 (21–72)
Mean BMI (kg/m^2^), SD	27.54 (±4.9)	28.3 (±5.4)	26.9 (±4.8)
Mean CTT baseline (°C), SD	19.5 (±5.2)	20.2 (±5.3)	17.3 (±6.2)
Mean duration of ColdU (month), SD	156 (±167.9)	84.3 (±167.7)	212.5 (±158.1)

### 4.2 Rilonacept treatment does not significantly reduce critical temperature thresholds (CTTs) in patients with cold urticaria

**FIGURE 2:**
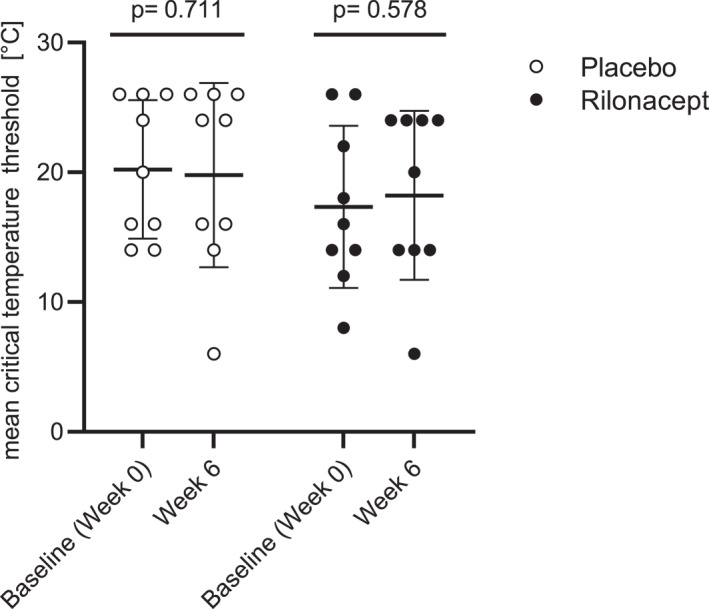
Changes in critical temperature thresholds (CTT) between baseline (week 0) and week 6 in placebo‐and rilonacept treated patients with ColdU. Rilonacept (160 mg) or placebo were injected in weekly intervals. CTT was determined every 2 weeks using TempTest^®^ device. Displayed are mean values and standard deviations. Differences were assessed by a paired *t*‐test.

Baseline mean CTTs were 17.3°C (SD: 6.2°C) in the rilonacept group and 20.2°C (SD: 5.3°C) in the placebo group. Mean CTTs did not change significantly during the 6‐week double‐blind phase, neither with placebo (CTT reduction of 0.45°C; *p* = 0.711), nor with rilonacept (CTT increase of 0.89°C, *p* = 0.578) (Figure 2).

### 4.3 Rilonacept treatment temporarily improves quality of life in cold urticaria patients

**FIGURE 3:**
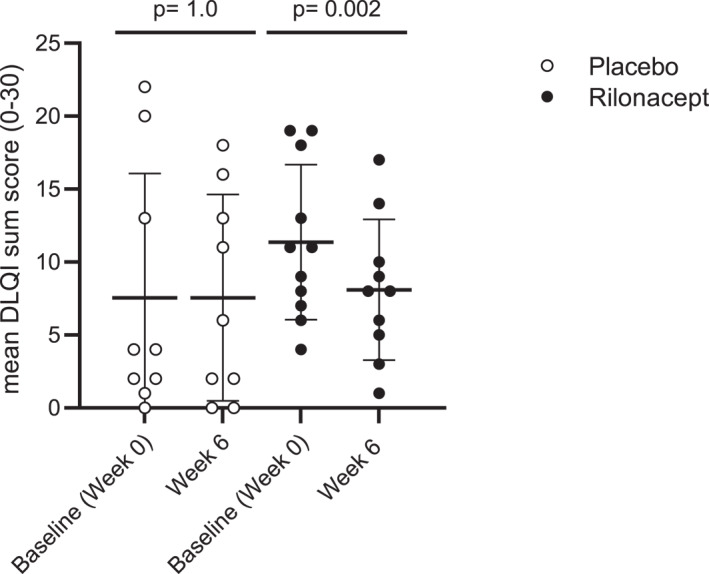
Changes in DLQI values (0–30) between baseline (week 0) and week 6 in placebo‐and rilonacept treated patients with ColdU. Rilonacept (160 mg) or placebo were injected in weekly intervals and quality of life was determined every two weeks by DLQI. Displayed are mean values and standard deviations. Differences were assessed by a paired *t*‐test.

Patient‐reported quality of life significantly improved in the rilonacept group (mean DLQI at week 0: 11.9 and after 6 weeks: 8.1; *p* = 0.002), but not in the placebo group (mean DLQI for baseline 7.5 and after 6 weeks 7.5; *p* = 1.0) (Figure 3). Within its subdomains, DLQI scores for daily activities and leisure time improved significantly (*p* = 0.01–0.037) in the rilonacept group.

### 4.4 Rilonacept treatment does not change serum IL‐6, IL‐18 and HSP‐70 levels in patients with cold urticaria

**FIGURE 4:**
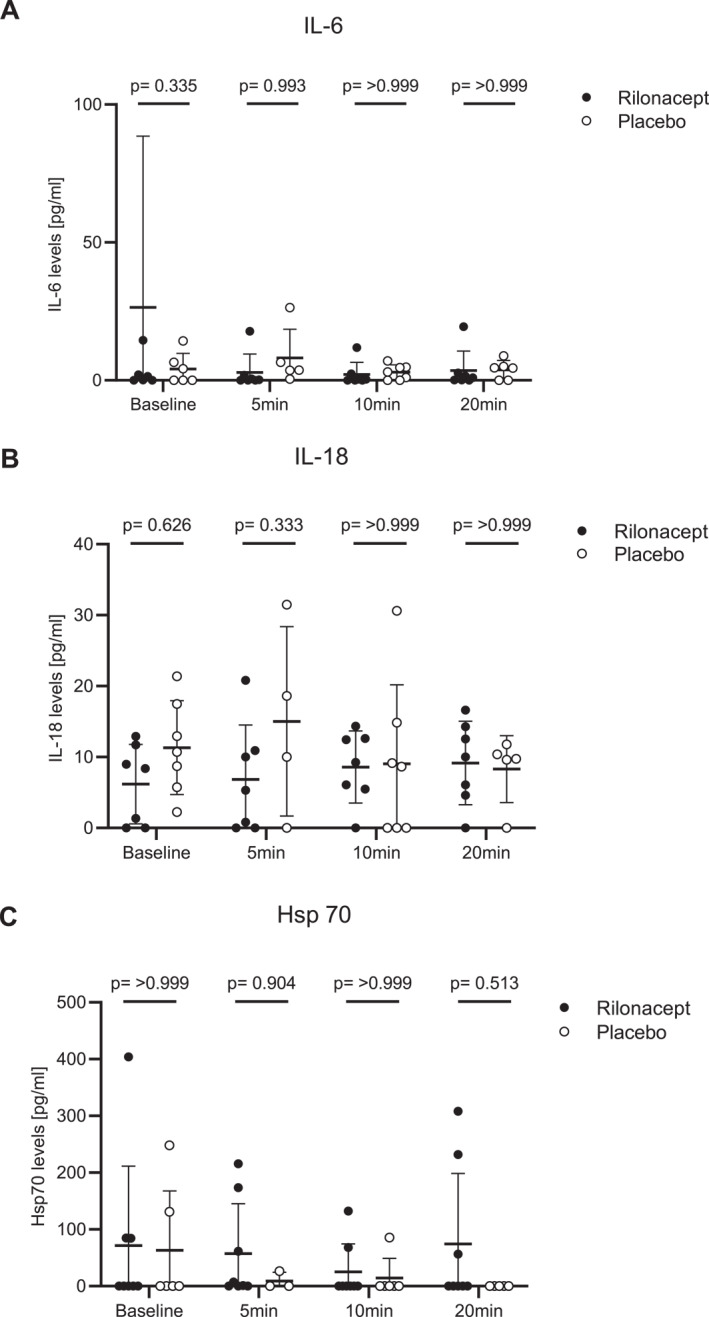
(A) Serum Interleukin (IL)‐6, (B) IL‐18 and (C) heat schock protein (HSP) 70 levels during cold water bath provocation were determined by ELISA. Displayed are mean values and standard deviations. Significances were assessed by a paired *t*‐test.

Post provocation serum levels of the inflammatory mediators IL‐6, IL‐18 and HSP‐70 showed high interindividual variability. None of them were significantly different in patients treated with placebo (*n* = 7) versus rilonacept (*n* = 7), (Figure 4A–C). Serum IL‐1RA levels were not detectable.

### 4.5 In ColdU, treatment with rilonacept does not affect clinical and serum inflammatory markers during the open‐label phase

Comparing baseline (week 0) with the end of the rilonacept open‐label treatment (week 12), there were no significant changes in CTTs (*p* = 0.453). Also, no significant changes of serum inflammatory markers IL‐6, IL‐18 and HSP‐70 before and after hand cold water bath provocation were observed at week 12. Although DLQI values improved in *n* = 6/11 patients at week 12 (*n* = 5/6 patients improved in the subdomains daily activities as well as symptoms and feelings) as compared to baseline, we could not observe a significant change during the open‐label treatment (*p* = 0.807) (Supplements Table S1–S2).

### 4.6 Rilonacept treatment is generally safe in cold urticaria

We observed 67 AEs in 17 patients throughout the study. One AE occurred during the screening period, 44 during the double‐blinded placebo‐controlled part (15 in the placebo group, 29 in the rilonacept group), and 22 during the open label treatment part (18 in the rilonacept 160 mg group, 4 in the rilonacept 320 mg group) of the study. No serious AEs were reported during the study, and there were no suspected unexpected serious adverse reactions. The AEs were mild or moderate. Most common AEs included cephalgia (*n* = 5) and infections of the upper respiratory tract (*n* = 3) in the placebo group. In the rilonacept treatment group, most common AEs were injection site reactions (*n* = 20 for 160 mg, *n* = 3 for 320 mg), infections (*n* = 4 infections of the upper respiratory tract, *n* = 2 infections of the urinary tract), cephalgia (*n* = 6) and fatigue (*n* = 3).

There were no clinically significant changes in routine laboratory parameters during the course of the study. Injection site reactions were the most common AEs reported with rilonacept treatment. Across the 20 patients treated with rilonacept, 8 (40%) demonstrated rilonacept‐specific low‐titer antibodies on at least one occasion. Development of anti‐rilonacept antibodies did not correlate with higher numbers of AEs or clinical responses.

## 5 DISCUSSION

The results of our study show that ColdU does not improve with anti‐IL‐1 treatment. Of note, previous studies had suggested that IL‐1 may possibly contribute to the pathogenesis of urticaria,^32^, ^33^ and a previous case report demonstrated complete remission of ColdU in response to treatment with the IL‐1 receptor antagonist anakinra.^26^ In contrast to these reports, our multi‐center, double‐blind, placebo‐controlled parallel group study with the IL‐1 inhibitor rilonacept suggests that IL‐1 is not a critical pathogenic driver of ColdU.

We observed that rilonacept treatment temporarily improved the quality of life in regard to leisure and daily activities as compared to placebo in patients with ColdU. This may be a true effect or coincidence. The facts that argue in favor of the latter include the lack of clinical efficacy, the absence of effects on other aspects of quality of life impairment, and the low numbers of patients assessed.

Antihistamine treatment (bilastine 80 mg/day) significantly reduced cutaneous levels of IL‐6 and IL‐8 after cold stimulation in a previous study in patients with ColdU.^14^ In contrast, rilonacept treatment, in the present study, had no effect on serum concentrations of IL‐1RA, IL‐6, IL‐18 and HSP‐70 in patients with ColdU. As we did not assess the cutaneous cytokine concentrations in this study, a direct comparison, however, is not possible.

Interestingly, upregulation of IL‐6 and IL‐1 mRNA expression was detected in lesional skin and peripheral blood mononuclear cells from CSU patients, suggesting that these cytokines are involved in CSU pathogenesis.^34^ Conflicting data exist on the role of serum cytokines in CSU. One study showed increased serum levels of IL‐1β, IL‐6 and IL‐18,^34^, ^35^, ^36^, ^37^ whereas another study could not confirm elevation of serum IL‐6 levels in CSU patients.^38^ Of note, the anti‐IL‐1β antibody canakinumab was demonstrated to lack efficacy in treating patients with moderate to severe CSU in a Phase II randomized, double‐blind, placebo‐controlled study.^39^ Similar to ColdU, IL‐1β does not appear to be a crucial cytokine in the pathogenesis of CSU.^39^

Rilonacept treatment was generally well tolerated and no severe AEs were observed. No patient discontinued the study due to a treatment‐emergent AE. Injection site reactions and infections were the most frequent AEs in our study, which has been observed in earlier rilonacept studies in patients with CAPS as well.^22^

Some CAPS patients, treated with rilonacept, developed detectable low‐titer anti‐rilonacept antibodies. In our study, we could also show low‐titer anti‐rilonacept antibodies in 40% of ColdU patients. However, the clinical significance of anti‐rilonacept antibodies is uncertain, as rilonacept‐specific antibodies did not affect the efficacy in CAPS patients.^22^

Limitations of our study comprise the relatively small patient number, particularly with regard to the open label treatment phase of the study and the missing analyses of skin mediators as well as the patient selection as patients with atypical ColdU (defined as patients with an atypical cold stimulation test [negative cold stimulation test at 4°C or ice cube test] and/or an atypical urticarial response e.g. systemic atypical cold urticaria or localized cold urticaria) were excluded. It remains open whether IL‐1 blockade may be a therapeutic target in patients with atypical ColdU. Cold urticaria, in patients with atypical variants, is clearly different from typical cold urticaria and this may be, at least in part, due to differences in the pathogenic drivers involved. Also, patients with atypical cold urticaria may present similar clinical features as patients with interleukin‐1‐mediated cold‐induced whealing due to autoinflammatory syndromes.^8^ In addition, we did not use the Cold Urticaria Activity Score (ColdUAS), a patient‐reported outcome measure to assess ColdU disease activity, as it was not available at the start of our study.^40^

## 6 CONCLUSION

In conclusion, this study shows that rilonacept treatment of ColdU patients does not reduce disease activity although it temporarily improved quality of life. For the future, further research on the clinical characterization, treatment response and pathophysiology of ColdU is crucial in order to better understand disease mechanisms and to identify potential therapeutic targets. Given the clinical heterogeneity of ColdU, it is particularly useful to stratify patients with ColdU according to clinical phenotypes and, possibly in the future, also according to pathophysiological endotypes, in order to develop individualized targeted therapies.

## AUTHOR CONTRIBUTIONS

**Hanna Bonnekoh**: Data curation, Formal analysis, Investigation, Writing ‐ original draft. **Monique Butze**: Data curation, Formal analysis, Writing ‐ original draft. **Sebastian Spittler**: Investigation, Methodology, Data curation, Formal analysis, Writing ‐ review and editing. **Petra Staubach**: Investigation, Methodology, Writing ‐ review and editing. **Karsten Weller**: Methodology, Writing ‐ review and editing. **Jörg Scheffel**: Methodology, Writing ‐ review and editing. **Marcus Maurer**: Data curation, Resources, Writing ‐ review and editing. **Karoline Krause**: Conceptualization, Formal analysis, Funding acquisition, Methodology, Project administration, Writing ‐ original draft.

## CONFLICT OF INTEREST STATEMENT

Hanna Bonnekoh received honoraria (advisory board, speaker) from AbbVie, Intercept Pharma, Novartis, Sanofi‐Aventis and Valenza Bio Inc. Outside the submitted work. Monique Butze has no conflict of interest. Sebastian Spittler has no conflict of interest. Petra Staubach received honoraria (advisory board, speaker) from AbbVie, Allergika, Almirall‐Hermal, Amgen, Beiersdorf, Biocryst, BMS, Boehringer‐Ingelheim, Celgene, CSL‐Behring, Eli‐Lilly, Galderma, Hexal, Janssen, Klinge, LEO‐Pharma, LETI‐Pharma, L´Oreal, Novartis, Octapharma, Pfizer, Pflüger, Pharming, Pierre Fabre, Regeneron, Takeda, Regeneron, Sanofi‐Genzyme und UCB Pharma outside the submitted work. Karsten Weller is or recently was a speaker and/or advisor for and/or has received research funding from BioCryst, CSL Behring, Moxie, Novartis, Shire/Takeda, and Uriach. Jörg Scheffel has conducted studies for, received research funds/was advisor for Allakos, Ascilion, AstraZeneca, CSL Behring, Escient, LeoPharma, Novartis, Sanofi, Third Harmonic Bio, ThirdRock, ThermoFisher, all outside the submitted work. Marcus Maurer is or recently was a speaker and/or advisor for and/or has received research funding from Astria, Allakos, Alnylam, Amgen, Aralez, ArgenX, AstraZeneca, BioCryst, Blueprint, Celldex, Centogene, CSL Behring, Dyax, FAES, Genentech, GIInnovation, GSK, Innate Pharma, Kalvista, Kyowa Kirin, Leo Pharma, Lilly, Menarini, Moxie, Novartis, Pfizer, Pharming, Pharvaris, Roche, Sanofi/Regeneron, Shire/Takeda, Third Harmonic Bio, UCB, and Uriach. Karoline Krause is or recently was a speaker and/or advisor for, and/or has received research funding from Berlin Chemie, Novartis and Takeda outside the submitted work.

## Supporting information

Supporting Information S1

## Data Availability

All datasets generated for this study are available from the corresponding author upon reasonable request.
