# Supplementary material for: Inhibition of interleukin‐1 with rilonacept is not effective in cold urticaria—Results of a randomized, placebo‐controlled study
Source: Clin Transl Allergy. 2023 Feb 28;13(3):e12226. doi: 10.1002/clt2.12226 (PMC9975456; doi:10.1002/clt2.12226)
Supplement: Supplementary file 1 — Supporting Information S1 [file CLT2-13-e12226-s001.pdf]

Supplements:

Table 1:

	**Baseline**	**Rilonacept (160 or 320 mg*) week 12**	**P-Value**
**Mean CTT, (°C), SD**	19 (±6.3)	18 (±7.2)	0.453
**Mean DLQI, sum score, SD**	6.82 (±6.9)	5.73 (±6.9)	0.311

Table 2:

**Rilonacept (160 mg or 320*) week 12**	**0 min**	**5 min**	**10 min**	**20 min**
**HSP70 levels, SD**	122.51 (±245.0)	46.48 (±103.9)	69.18 (±154.7)	29.19 (±65.3)
**IL-18 levels, SD**	11.01 (±3.6)	18.63 (±13.2)	16.74 (±11.7)	8.29 (±6.3)
**IL-6 levels, SD**	4.21 (±2.9)	5.03 (±3.8)	5.69 (±4.2)	4.39 (±2.2)

Supplements:

Table 1: Results of the open-label treatment phase: Changes in critical temperature thresholds (CTT) and in Dermatology Life Quality Index (DLQI) values between baseline (week 0) and week 12 in rilonacept treated patients with ColdU. *Rilonacept 160 mg n=9 patients, Rilonacept 320 mg n=2 patients. IL Interleukin, SD Standard deviation

Table 2: Results of the open-label treatment phase: serum Interleukin (IL)-6, IL-18 and heat schock protein (HSP) 70 levels during cold water bath provocation determined by ELISA. *Rilonacept 160 mg n=3 patients, Rilonacept 320 mg n=2 patients. SD Standard deviation
